# Primary congenital hypothyroidism: a clinical review

**DOI:** 10.3389/fendo.2025.1592655

**Published:** 2025-08-05

**Authors:** Paolo Cavarzere, Valentina Mancioppi, Riccardo Battiston, Valentina Lupieri, Anita Morandi, Claudio Maffeis

**Affiliations:** ^1^ Department of Mother and Child, Pediatric Unit B, University Hospital of Verona, Verona, Italy; ^2^ Department of Surgery, Dentistry, Gynecology and Pediatrics, Section of Pediatric Diabetes and Metabolism, University of Verona, Verona, Italy

**Keywords:** congenital hypothyroidism, thyroid gland, newborn screening for congenital hypothyroidism, dyshormonogenesis, dysgenesis

## Abstract

Congenital hypothyroidism (CH) is the most common neonatal endocrine disorder. It is one of the clinical conditions that has benefited most from the introduction of newborn screening 50 years ago, as clinical management has changed and long-term consequences have been significantly reduced. In areas where neonatal screening is active, most affected patients show a clinically normal phenotype and/or only mild symptoms. At the same time, thanks to a progressive reduction in the TSH level used as cut-off for neonatal screening, the number of cases of CH with gland *in situ* is increasing, while the number of patients with abnormal thyroid development has remained essentially unchanged over time. Furthermore, important changes are observed in managing patients with CH and gland *in situ*. On the one hand, they are subjected to genetic investigations to understand the underlying molecular mechanism; on the other hand, a reassessment of thyroid function is suggested starting from the sixth month of life if their L-thyroxine requirement is low. This review aims to describe the clinical approach to CH and to optimize the management and treatment of this disease.

## Introduction

Hypothyroidism is a disease characterized by low circulating levels of thyroid hormones (TH), which are inadequate to exert their metabolic and neurological effects at the cellular level. It is caused by a defect at any level of the hypothalamic-pituitary-thyroid axis, resulting in the inability to produce TH in sufficient quantities. When the defect is present at birth, the condition is defined as congenital hypothyroidism (CH). Primary CH (due to a thyroid gland defect) is classified as severe, moderate, or mild based on blood concentrations of FT4: <5 pmol/L, 5–10 pmol/L, and 10–15 pmol/L, respectively (1). Furthermore, hypothyroidism can be secondary (due to a TSH deficiency), tertiary (due to a TRH deficiency), or resulting from tissue resistance to the action of TH, a condition known as peripheral hypothyroidism (2).

Congenital hypothyroidism is the most common neonatal endocrine disorder. In addition, it is one of the clinical conditions that has benefited the most from the introduction of newborn screening 50 years ago, since clinical management has profoundly changed and long-term consequences have been significantly reduced. In areas where newborn screening is active, most affected patients show a clinically normal phenotype and/or only mild symptoms. At the same time, thanks to a progressive reduction in the TSH level used as cut-off for neonatal screening, the number of cases of CH with gland *in situ* is increasing. However, only 30% of the countries in the world have activated newborn screening for CH, so neonatal screening remains an open question still unresolved. Furthermore, the progress in genetic and epigenetic studies allows a more accurate genotype-phenotype characterization, leading to the identification of new variants and new phenotypes associated with known variants. In this context, the role of the environmental factor in the genetic expression of the identified variant may be important. Finally, the treatment of CH in paediatric age still represents a challenge, particularly regarding the initial dosage, the most appropriate pharmacologic formulation, and the most appropriate time to attempt a possible suspension of treatment.

This narrative review aims to describe the clinical approach to CH and to optimize the management and treatment of this disease.

## Background: anatomy, embryogenesis and function

The thyroid is a bilobed gland located in the neck region, composed of two types of cells: follicular cells, which produce thyroxine, and parafollicular cells, which produce calcitonin. Follicular cells constitute the predominant cell population and are organized into thyroid follicles (3, 4).

Thyroid embryogenesis begins between the 20^th^ and 22^nd^ days of fetal development, with the appearance of the thyroid bud as a thickening of the pharyngeal floor. On the 26^th^ day of development, the thyroid diverticulum begins its migration to its definitive pretracheal position, reached between the 48^th^ and 50^th^ day of gestation. The formation of thyroid follicles and gene expression involved in hormonogenesis begin at the end of migration. Hormone synthesis begins between the 10^th^ and 12^th^ week of development, incorporating iodine into TH (5–8).

The main function of the thyroid gland is the production of TH. In early gestation, the fetus depends entirely on maternal TH transfer; consequently, total serum T4 and T3 levels are low and entirely dependent on placental function and maternal thyroid status. From the 20^th^ week of gestation, the fetal hypothalamic-pituitary-thyroid axis begins to function, and fetal TSH and T4 production progressively increases (9). Plasma TH levels correlate with gestational age; consequently, premature newborns have lower T4 and FT4 values than term infants (10–13).

When the fetus has an abnormal thyroid ontogeny, transplacental TH transfer during gestation is sufficient to preserve fetal brain development and function (9, 14, 15). In contrast, in the presence of both maternal and fetal hypothyroidism, significant impairment of neurointellectual development occurs despite the administration of adequate therapy immediately after birth (14, 16). Maternal hypothyroidism during the first month of gestation can lead to mild but significant cognitive impairment in the offspring (17, 18).

At birth, the rapid fall in ambient temperature causes a TSH peak within 30 minutes of birth, with a consequent increase in FT4 levels. In term newborns, FT4 levels decrease within 4–6 weeks of life, whereas in those born prematurely, the increase is attenuated and related to gestational age. In contrast, preterm infants born before 30 weeks or with low (LBW) or very low birth weight (VLBW) show a decline in FT4 levels with a nadir at 1–2 weeks of life, unaccompanied by an increase in TSH (12, 19).

## Epidemiology

Congenital hypothyroidism is the most frequent endocrine disorder in newborns. The incidence of CH has changed over the years. Before the start of newborn screening for this disease in 1974, the incidence of CH was estimated at 1:7,000; subsequently, it progressively doubled to reach 1:3,500 live births in regions with sufficient iodine availability (20, 21). In the last two decades, the incidence of CH has doubled again. A possible explanation for the increase in CH incidence is the change in the screening strategy with a progressive reduction of the TSH value used as a cut-off, which has increased the sensitivity of the screening test (22). Furthermore, greater attention to particular categories of newborns considered at high risk of CH, such as twins, LBW and premature newborns, also increasing due to the progress of perinatal medicine, could have contributed to an increase in CH detection. Following these hypotheses, the number of newborns with CH due to athyreosis seems unchanged, while there is a clear increase in newborns with CH and gland *in situ*. Finally, other causes of this increased incidence could be the change in the population’s ethnicity subjected to screening and the presence of environmental and genetic factors (23). **Table 1** shows the change in the incidence of CH in different geographic areas over time (24, 25).

**Table 1:** Modification of incidence of CH in different geographic areas over time.

Country	Incidence in 1980-1990 years	Incidence in 2000 years
USA	1:3,985	1:2,273
New York State	1:3,373	1:1,415
Nord-Est Italy	1:3,297	1:1,822
Australia	1:5,747	1:2,825
Lombardy (Italy)	1:2,654	1:1,154
United Kingdom	1:2,702	1:1,078
Greece	1:3,384	1:1,749
Italy	1:3,150	1:2,350

The data highlighted in grey are previous unpublished data from our case history.

Congenital hypothyroidism is more common in females than in males (2:1 ratio) and the Hispanic population than in Afro-ethnic individuals (26). In addition, children with Down syndrome have a higher risk of developing CH (27).

## Aetiology and pathogenesis

The hypothalamic-pituitary-thyroid axis maintains stable FT4 concentration in normal subjects; on the contrary, in children with CH there is a hypo-function of the gland with an increase in TSH concentrations, an attempt to compensate for the hormonal deficit. Exceptions to this mechanism are central hypothyroidism or other rarer conditions.

The most frequent cause of primary CH is thyroid dysgenesis, which includes athyreosis due to the complete absence of thyroid tissue, ectopic gland due to anomalous migration of the embryonic thyroid, and thyroid hypoplasia or hemithyroid/single lobe due to defective growth of the gland after complete migration (28–31). Most patients with ectopic thyroid have a gland located on the dorsum of the tongue or, less frequently, sublingually (30, 32, 33).

The exact aetiology of thyroid dysgenesis is unknown. It may be associated with *TSHR* mutations or mutations in the genes of transcription factors that regulate thyroid development (such as *Nkx2.1/Ttf-1, Foxe/Ttf-2, Nkx2-5, Glis3*, and *Pax-8*) (34–36). A genetic condition in thyroid dysgenesis has been found in less than 5-10% of cases, while familial status is present in about 2% (37).

In the remaining cases, the thyroid gland is *in situ* and, in <50% of these conditions, the disorder is related to inborn errors of metabolism during one of the several steps required for normal TH synthesis (thyroid dyshormonogenesis) (28, 38). Typically, thyroid dyshormonogenesis is inherited in an autosomal recessive manner and mutations in the thyroid peroxidase (*TPO*) gene are the most common causes of inherited defects in CH. Patients with dyshormonogenesis have defects in one of the critical steps in TH synthesis. All these defects typically have an autosomal recessive transmission mode and are generally not associated with other malformations, except Pendred syndrome, in which CH is related to sensorineural hearing loss (39–43). In some cases, dual oxidases 2 (*DUOX2)* or *DUOX1* are inherited in an autosomal dominant manner. The different genetic causes of thyroid dyshormonogenesis can be evaluated and classified by thyroid scintigraphy with perchlorate test, measuring radioactive iodine uptake (44). In this way, the total iodine organification defect from the partial iodine organification defect can distinguished. Radioactive iodine uptake is little or absent in iodine uptake defects such as sodium iodide symporter (NIS)*/SLC5A5* variants. Partial or total defects in iodine organification due to mutations in *TPO, DUOX2*, *DUOXA2*, and *PENDRIN* genes present a radioactive iodine concentration of 10% to 90% and, therefore, a positive perchlorate test. Typically, patients with these variants present with a goiter (45). Recently, mutations in *SLC26A7* have been described as causing CH with a partial iodine organification defect and goiter (46). In contrast, the perchlorate test is negative for defects in thyroglobulin synthesis, storage and release or in *DEHAL1* variants. The latter case is characterized by a very rapid reduction in iodine fixation in the thyroid.

**Figure 1:**
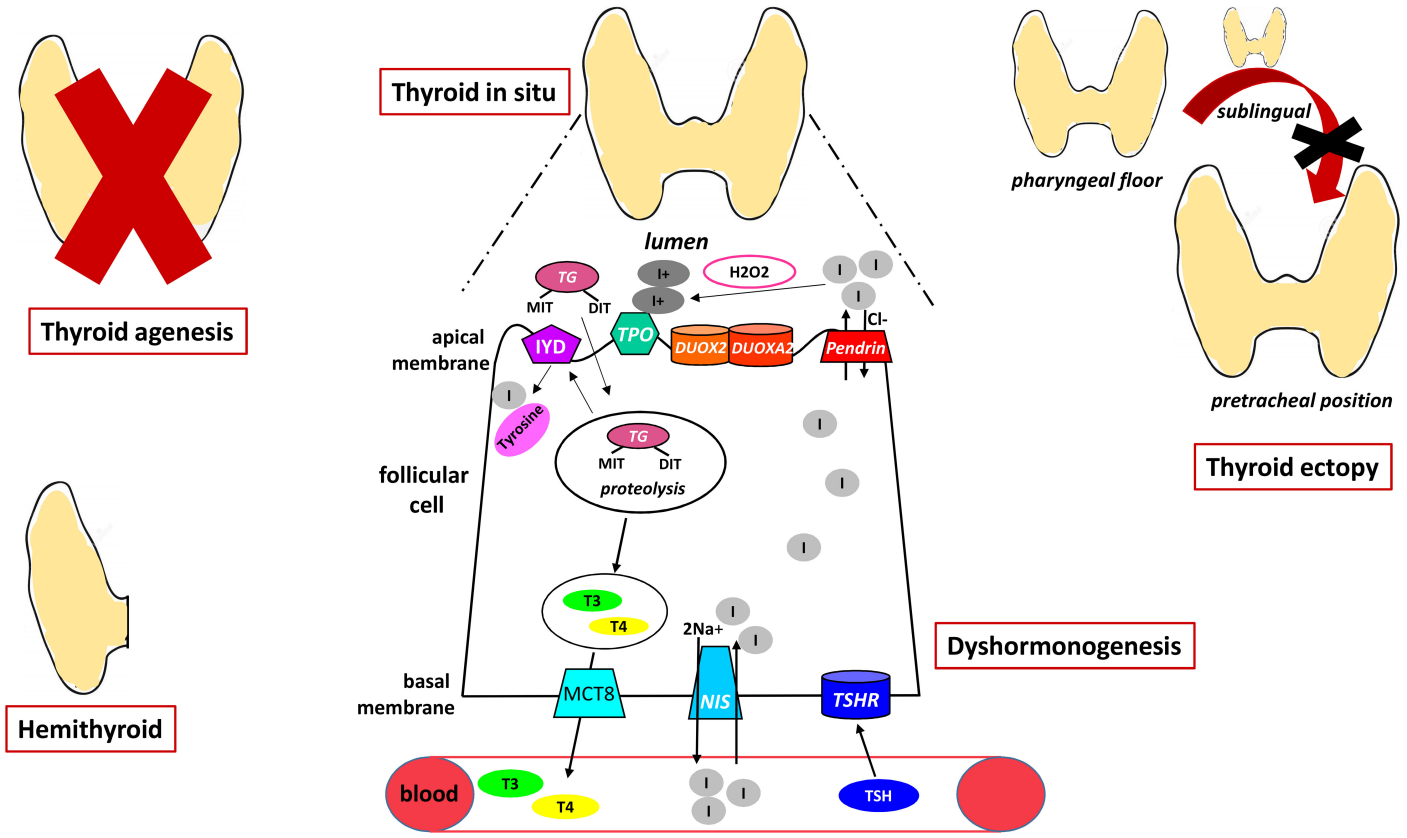
The figure shows the pathogenesis of primary CH representing the different forms of dysgenesis and the mechanisms underlying dyshormonogenesis. NIS, sodium iodide symporter; TSHR, TSH receptor; MCT8, monocarboxylate transporter 8; DUOX2, dual oxidases2; DUOX2A, dual oxidases2A; MIT, monoiodotyrosine; DIT, diiodotyrosine; TG, Tireoglobulin; IYD, dehalogenase 1; TPO, thyroid peroxidase.

New thyroid phenotypes have recently been described in known genes, such as thyroid ectopy in patients with *DUOX2* variants and gland *in situ* without goiter or thyroid hypoplasia in patients with *PENDRIN*, *DUOX2* or *TPO* variants. **Figure 1** summarizes the pathogenesis of CH.

**Table 2** summarizes the genes implicated in thyroid dysgenesis and dyshormonogenesis, their mode of transmission, and the clinical phenotype with which they are associated (35, 36, 39, 47–53).

**Table 2:** Genes associated to CH, in relation to its etiology.

Type of CH	Gene	Heredity	Phenotype	Associated diseases
Thyroid dysgenesis	*TSHR*	AD/AR	variable (from gland in situ to complete hypoplasia)	/
	*Nkx2.1* *(Ttf-1)*	AD	variable	neurological phenotype (choreoathetosis), respiratory distress
	*FOXE1* *(Ttf-2)*	AR	athyreosis, severe hypoplasia	cleft palate, choanal atresia, spiky hair
	*Nkx2-5*	?	gland in situ, variable	congenital heart disease
	*Glis3*	AR	variable (gland in situ, athyreosis)	neonatal diabetes, polycistic kidneys, cholestasis, dysmorphic facies, congenital glaucoma, skeletal abnormalities, learning difficulties
	*Pax-8*	AD	variable	renal and urogenital tract anomalies
	*JAG1*	AD	thyroid hypoplasia, variable	heart defect and hepatic cholestasis
	*NTN1*	?	thyroid ectopy	arthrogryposis, congenital heart disease
	*CDCA8* *(BOREALIN)*	AD/AR	thyroid ectopy, variable	congenital heart disease (?)
	*TUBB1*	AD	gland ectopy (mainly)	platelet hyperaggregation
	*TRPC4AP*	AD	gland hypoplasia	/
	*GBP1*	AD/AR	dysgenesis or normal gland	/
Dyshormonogenesis	*SLC5A5 (NIS)*	AR	goiter iodine uptake absent/- high thyroglobulin	late neurodevelopmental delay (rare)
	*SLC26A4 (PDS)*	AR	goiter (+/-) iodine uptake +, PIOD high thyroglobulin	Pendred syndrome (sensorineural deafness with enlarged vestibular aqueduct)
	*DUOX1* */DUOX2*		goiter (+/-); (ectopy) iodine uptake +, PIOD or TIOD high thyroglobulin	
	*DUOXA2*	AR	goiter iodine uptake +, PIOD or TIOD high thyroglobulin	
	*TPO*	AR	goiter (+/-) iodine uptake +, TIOD, high thyroglobulin	
	*TG*	AR	congenital or rapidly growing goiter iodine uptake +, perchlorate - low thyroglobulin	
	*IYD (DEHAL1)*	AR (incomplete penetrance)	goiter or normal gland iodine uptake =, perchlorate - high thyroglobulin MIT and DIT in serum and urine	late neurodevelopmental delay (rare)
	*SLC26A7*	AR	goiter iodine uptake =, PIOD high thyroglobulin	

AD, autosomal dominant; AR, autosomal recessive; PIOD, partial iodine organification defect; TIOD, total iodine organification defect; MIT, monoiodotyrosine; DIT, diiodotyrosine.

As reported in **Table 3**, thyroid dysgenesis often occurs in some syndromic conditions. In rare cases, a syndromic condition is associated with dyshormonogenesis.

**Table 3:** Syndromic conditions associated to congenital hypothyroidism and correlated genes.

Type of CH	Syndrome	Gene
Thyroid Dysgenesis	Brain-Lung-Thyroid syndrome	*Nkx2.1 (Ttf-1)*
	Di George syndrome	*TBX1*
	Bamforth-Lazaryus syndrome	*FOXE1*
	Williams-Beuren syndrome	*ELN*, *BAZ1B*
	Kabuki syndrome	*KMT2D*, *KDM6A*
	Down syndrome	*DYRK1A*
	Townes-Brocks syndrome	*SALL1*
	Johanson-Blizzard syndrome	*URB1*
	Ohdo syndrome	*KAT6B*
	Takenouchi-Kosaki syndrome	*CDC42*
	Blepharo-cheilo-dontic syndrome	*CDH1*, *CTNND1*
Dyshormonogenesis	Pendred syndrome	*SLC26A4/PDS*
	Pseudohypoparathyroidism	*GNAS*

It is important to recognize that CH may have a multifactorial origin, often resulting from the oligogenic basis of sporadic cases of CH. This can help explain the variable expressivity and penetrance of genetic abnormalities observed in several family cases of CH.

## Clinical

CH’s clinical signs and symptoms are the consequence of the absence of TH effects. At the cardiovascular level, TH reduces systemic vascular resistance and increases heart rate, contractility, and output; promotes renal salt and water excretion, stimulates gastrointestinal motility, increases basal metabolism and body temperature, and ultimately regulates growth and neurological development (54).

Transplacental passage of maternal TH protects newborns with CH for approximately two weeks (55); consequently, only 1-4% of infants with CH are diagnosed at birth by clinical examination (56). Newborns with CH are generally late-born, have a high birth weight for gestational age, and a large posterior fontanel (57). However, the clinical manifestation of CH is often subtle, nonspecific, and easily overlooked. Late signs of CH include prolonged jaundice, skin mottling, large anterior fontanel, abdominal distension, umbilical hernia, hypotonia, hypothermia, lethargy, bradycardia, macroglossia, constipation, feeding difficulties, failure to thrive, hoarse crying, hearing impairment, neurodevelopmental delay, and, in the absence of early diagnosis, irreversible intellectual disability as the most serious consequence (58, 59). Goiter, usually associated with dysormonogenesis, is rare in the neonate and often appears only in late childhood (56). These symptoms are more severe in neonates with dysgenesis (60, 61).

Furthermore, CH patients have higher neonatal morbidity and congenital malformations than matched controls (59, 62). Cardiac malformations are the most frequent congenital defects associated with thyroid dysgenesis, although the influence of cardiac development on thyroid organogenesis has not yet been demonstrated (63).

Recently, extrathyroidal congenital anomalies involving the cardiac system, urogenital tract, gastrointestinal tract, and musculoskeletal system have been identified (64). These defects are mainly observed in patients with thyroid agenesis and dyshormonogenesis.

Finally, it has been shown that the cumulative incidence of neurological disorders is higher in patients with CH (59).

## Newborn screening

The first newborn screening programs for CH were developed 50 years ago, in 1974, and have now been successfully implemented in most of the world (North America, Western Europe, Japan, Australia, part of Eastern Europe, Asia, South and Central America) (65–67). The main goal of CH screening is the eradication of intellectual disability due to CH, and in this regard, the costs of screening are significantly lower than those of treatment in case of missed CH diagnosis during newborn screening.

In this context, one important challenge is the extension of newborn screening in the areas of the world where it is still lacking. The extension of the screening will reduce the costs of disability for patients who are not diagnosed at birth and allow otherwise healthy children to live with dignity also in non-industrialized areas.

As a screening method, it can be used as a T4 primary strategy or, more frequently, as a primary TSH strategy or as a combined primary approach, which is the ideal one (68–70). Today, 50 years after the beginning of newborn screening, only a few countries worldwide have a screening program capable of detecting central CH (71). Although this is not the subject of this review, it is worth mentioning because it is a more frequent condition than previously thought and is the only pituitary deficiency detectable by newborn screening.

Screening should be performed between 48 and 72 hours of life, in any case before discharge from the nursery, which in some cases occurs earlier, at 24 hours of life. It has been suggested that the screening test should be delayed because, as explained above, there is a physiologic increase in TSH secretion in the first hours after birth, which slowly decreases in the following hours and may give false positive screening results (72). The screening test must be performed before a blood transfusion otherwise the diagnosis may be missed. In critically ill or preterm neonates, blood for screening should be drawn within 7 days of life. Neonates admitted to the Neonate Intensive Care Unit (NICU) usually present with more urgent medical problems, but, nevertheless, the sample should be collected before discharge or transfer to another hospital (73).

In some conditions, such as preterm infants, TSH elevation may occur later due to immature hypothalamic-pituitary axis function (69, 74, 75). To avoid false negative results, newborn screening with the TSH-based method should be repeated at 2–4 weeks of age in patients in high-risk patients, such as preterm and LBW newborns, critically ill newborns, twins, and newborns with trisomy 21 (2, 76, 77).

## Diagnosis

All newborns with abnormal newborn screening results should undergo serum FT4, FT3 and TSH measurements to confirm the diagnosis: newborns with hypothyroidism typically have low FT4 and high TSH concentrations (69, 75). It is imperative to perform TSH and FT4 measurements as soon as possible in all cases with clinical suspicion of hypothyroidism, regardless of screening results (69).

**Figure 2:**
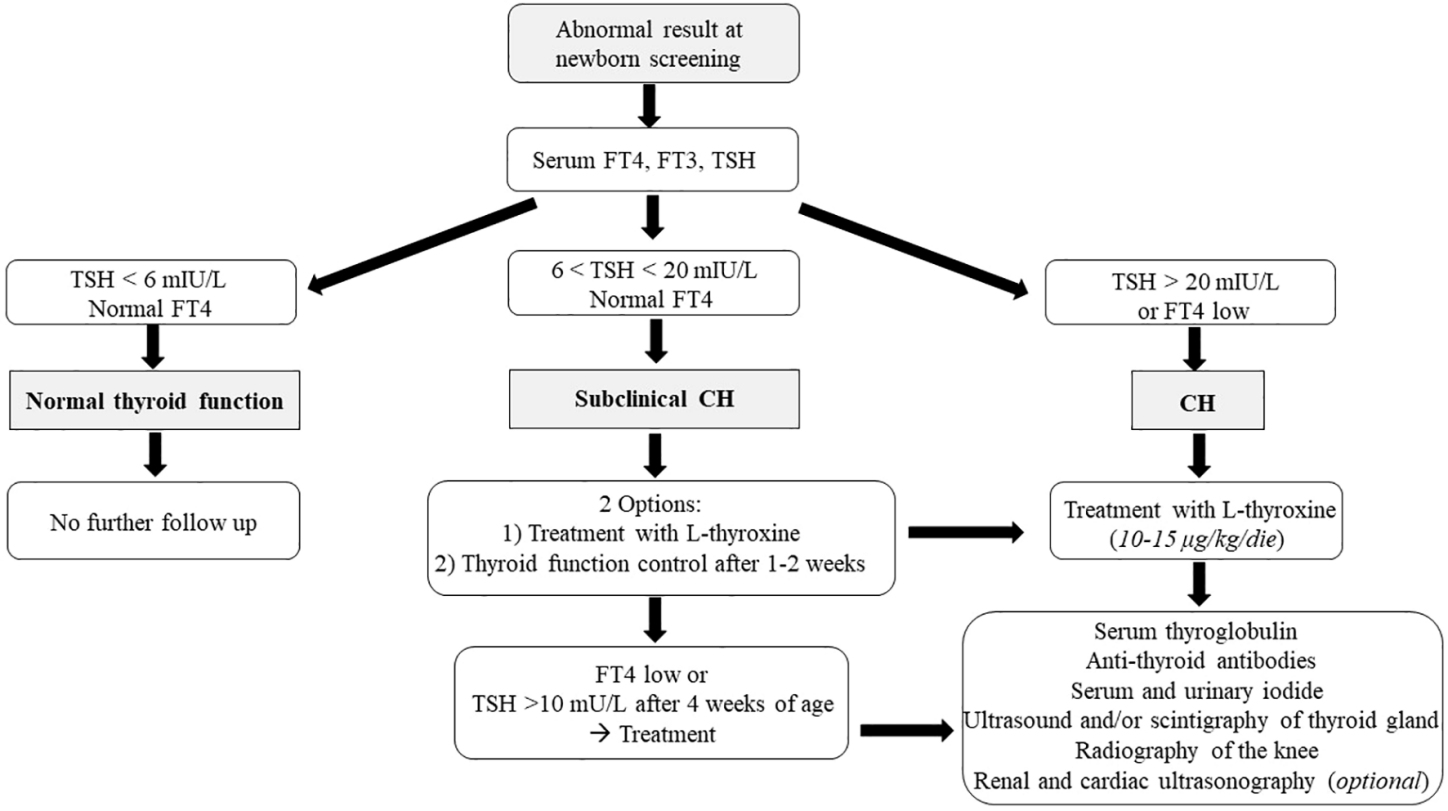
The figure shows the diagnostic algorithm of primary CH after the identification by neonatal screening.

If the TSH value is between 6 and 20 mIU/L and FT4 is within the normal range, subclinical CH should be considered, while it is unclear whether replacement therapy may be useful. In these cases, two different approaches are possible: the patient can start immediately L-thyroxine treatment, with subsequent reassessment of thyroid function, or treatment can be postponed, and thyroid function can be reassessed after 1–2 weeks (2). In any case, if during follow-up FT4 decreases or TSH elevation >10 mU/L persists beyond 4 weeks of age, treatment remains recommended (**Figure 2**) (78).

Conversely, treatment should be started immediately if FT4 concentrations are below the normal range and/or TSH levels are >20 mIU/L on confirmatory testing (**Figure 2**).

**Figure 3:**
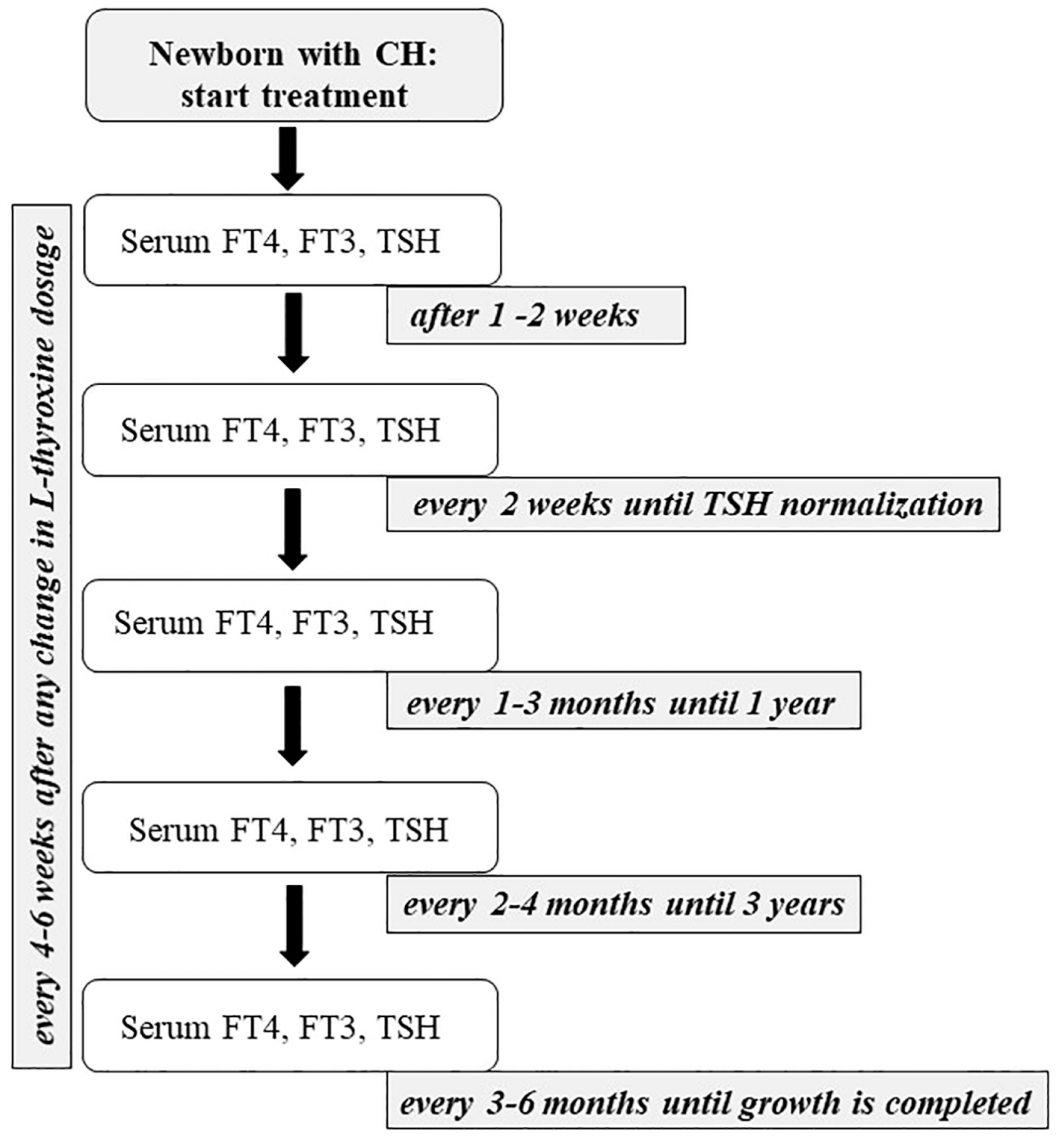
The figure shows the flow-chart describing the management of primary CH from the beginning of the treatment to the end of growth.

As shown in **Figure 3**, if CH is suspected, it is necessary to evaluate not only TH but also serum thyroglobulin and anti-thyroid antibodies. Elevated thyroglobulin levels may suggest dyshormonogenesis, and absent levels suggest athyreosis (56, 69, 79). Tests for thyroid autoimmunity (anti-peroxidase, anti-TSH receptor, and anti-thyroglobulin antibodies) allow CH to be associated with the trans-placental passage of maternal antibodies (80, 81). Finally, serum and urinary iodide measurements help to determine iodide deficiency or excess and consequently hypothesize a transient form of CH (82, 83).

The etiological diagnosis of CH is obtained by thyroid ultrasound and/or scintigraphy (44).

Thyroid ultrasound is useful in defining the size and structure of a normally positioned gland. It is operator-dependent and, therefore, less useful in making a diagnosis because it does not allow the diagnosis of an ectopic gland (84). On the other hand, fetal ultrasound is widely available, easy to perform, has a low cost, and can allow the physician to make a prenatal diagnosis of fetal dysgenesis or goiter (85). Thyroid volume in newborns ranges from 0.84 ± 0.38 to 1.62 ± 0.41 mL and remains stable during the first 3 weeks of life (86, 87).

Scintigraphy can be performed using 99m-Technetium (Tc-^99^) or Iodine-123 (I-^123^), both captured by NIS on the basal membrane of thyroid follicular cells (44). As shown in **Table 4**, I-^123^ is more expensive and requires delayed image acquisitions but less irradiating. It helps obtain information on the functionality of the thyroid gland. However, more frequently, scintigraphy is performed with Tc-^99^ (19, 88, 89). Scintigraphy allows the diagnosis of an ectopic gland and the exact identification of the contrast area. In these cases, it is possible to define a permanent CH (90). In other cases, the absence of Tc-^99^ or I-^123^ allows the diagnosis of athyreosis or, in the presence of high doses of thyroglobulin, the diagnosis of mutations that inactivate the TSH receptor, NIS mutation, previous exposure to iodine or maternal anti-TSH antibodies (91). When scintigraphy with I-^123^ shows a normally located thyroid, a positive perchlorate discharge test will allow, in most cases, the diagnosis of iodine organification defects (92). This test allows us to evaluate whether the thyroid follicular cells can retain iodine. It is considered positive if, after 1 hour, the radioisotope discharge is greater than 10% of the administered dose.

**Table 4:** Characteristics of the 2 tracers commonly used in clinical practice.

Advantages	Radioactive Tracer	Disadvantages
More available Less expensive Faster Shorter half-life	**^99m^Tc** (*ev*)	Not organified Low quality Higher radiation dose (*50-250 µCi/kg*)
Higher contrast Organization process Perchlorate test Low radiation dose (*3-10 µCi/kg*)	**^123^I** (*os*)	Less available More expensive Slow Long half-life

Tc-^99^, 99m-Technetium; I-^123^, Iodine-123.

For a post-natal diagnosis of CH, the best approach is to perform both an ultrasound scan, which will allow to determine the size and morphology of the thyroid, and a scintigraphy, which can detect the functionality of the thyroid tissue and obtain a perchlorate discharge test for iodine organification defects (93).

When diagnosing CH, a knee x-ray is useful since the absence of epiphyses indicates the fetal origin of the disease and, therefore, indicates that the child is at increased risk of developmental delay (94). Heel radiographs can be used for the same purpose.

Renal and cardiac ultrasounds are optional investigations to evaluate associated malformations, such as cardiac septal defects, the most frequently associated heart diseases (34).

At the time of diagnosis, providing parents with additional written information about their children’s medical condition and the care they require is useful.

## Genetic

Recent advances in genetic techniques using next-generation sequencing (NGS) and whole-exome or whole-genome sequencing have provided new insights into the genetics of CH, with the identification of new candidate genes and novel thyroid phenotypes associated with known mutations. Additionally, knowledge of a specific genotype may, in the long term, influence the treatment and management of these conditions.

Genetic testing aims to improve CH’s diagnosis, treatment, and prognosis. It is recommended in patients with dysormonogenesis or familial dysgenesis. For some specific patients with CH, genetic counseling should be offered to explain heredity and risk of recurrence. In particular, it is aimed at children with deafness or family history of deafness, neurological signs such as hypotonia, choreoathetosis and intellectual disability, lung disorders, congenital heart disease, cleft palate, renal malformations, and in the presence of signs of Albright hereditary osteodystrophy (2, 95). Preferably, genetic analysis should be performed using array CGH, NGS of gene panels, or WES. Recent research has shown that, using NGS in patients with gland *in situ*, a molecular explanation is more frequent in children with severe forms of CH (such as TSH ≥ 80 mUI/L or FT4 ≤ 5 pmol/L at diagnosis) (96).

Finally, environmental factors, such as iodine, may play an important role in the genetic expression of the identified variant. Further studies in this field could contribute to understanding the different clinical phenotypes related to the same variant and improve the management of this clinical condition.

## Treatment and management

The treatment of CH has been known for a long time, however, it has changed over time, and its management will remain challenging in the coming years. Further studies will concern the treatment required by some specific genetic conditions, the use of different, but no less effective, therapeutic options, such as the new formulations in drops or vials, in addition to the better-known tablets, and finally, the duration of therapy both in transient forms and in some genetic forms of CH.

Treatment with L-thyroxine should be started as soon as possible and no later than 2 weeks after birth (2, 97). The aim of therapy is to normalize T4 levels within 2 weeks and TSH values within 1 month (61). In this way, it is possible to ensure the normal growth and development of children with neurocognitive outcomes similar to the child’s genetic potential (55, 69). An initial dosage of 10-15 μg/kg/day is recommended to normalize TSH rapidly (69, 98). In general, patients with severe CH require higher doses than patients with mild CH, and the latter require higher doses than children with CH and normal FT4 concentrations before treatment. Based on the positive ino- and chronotropic effect of L-T4, it is recommended to use a lower initial dose, approximately 50% of the usual dose, in children with congenital heart disease and impending heart failure. Subsequently, the dose may be increased based on serum FT4 and TSH levels (2).

L-thyroxine should be administered orally, once daily, if possible, at the same time each day, and it is recommended to use the branded formulation rather than the generic one (2). It is available in several dosage forms such as tablets (the form available in most countries), soft capsules (containing L-thyroxine dissolved in water and glycerine preserved in a gelatinous matrix to protect the hormone from degradation), liquid preparation, drops, and in some countries also as intravenous solution (36).

If intravenous treatment is necessary, the initial dose should not exceed 80% of the oral dose, subsequently, the dose will be adjusted by measuring serum TSH and FT4 (2).

The therapy should be taken on an empty stomach, in the morning, with a differentiated administration time based on the formulation. For solid formulations, it is recommended to administer the drug at least 30 minutes before breakfast to optimize its absorption, while for liquid formulations, a shorter time interval before food intake is acceptable, due to their different pharmacokinetics. It is absorbed primarily in the proximal small intestine so that untreated celiac children may have reduced absorption and, consequently, a smaller effect (2). L-thyroxine tablets should be crushed and mixed with a few millilitres of liquid; however, they should not be mixed with bottles of milk that infants may not finish. Several substances such as soy protein (present in soy-based formulas), iron, and calcium have been reported to interfere with thyroxine (55, 56, 69). Although some liquid solutions of L-thyroxine contain ethanol, they also appear safe in the paediatric population, allowing normal growth and neuromotor development (99–101). In addition, liquid formulations are absorbed more rapidly with a low risk of drug-food interactions (102). A possible overtreatment during the first month of therapy has been described, therefore, lower doses and a more rigorous follow-up in the initial phase are suggested. A new liquid formulation without ethanol has recently been proposed, with good results (100, 103, 104).

So far, no advantages have been demonstrated in the association of L-thyroxine with triiodothyronine treatment (78, 102).

As represented in **Figure 3**, infants with CH should be evaluated 1 (if treatment is started with a higher dose of L-thyroxine) or 2 weeks after initiation of therapy, then every 2 weeks until complete normalization of serum TSH, then every 1 to 3 months for the first 12 months of life. Between 1 and 3 years, it is suggested to continue clinical and biochemical follow-up evaluations every 2–4 months. Thereafter, a check-up is necessary every 3 to 6 months until growth is completed (2). During follow-up, the drug dosage should be modified based on TSH and FT4 levels, especially during the first 3 years of life when growth is most rapid (69). Furthermore, TSH levels should be maintained within the age-specific normal range during the first year of life. Although some authors suggest maintaining TSH between 0.5 and 2 mU/mL, with FT4 concentrations in the upper half of the reference range, no convincing data supports these limits. If the TSH is within the normal reference range, an FT4 concentration above the upper limit of the reference range may be acceptable, and the same dose of L-thyroxine may be recommended. There is no evidence of a possible adverse effect of overtreatment if this is limited to short periods. Consequently, a reduction in the dose of L-thyroxine is suggested not only when the patient shows a single elevated FT4 value, but only when TSH is suppressed, in the presence of symptoms of hyperthyroidism or after a second measurement of elevated FT4 (2). Serum FT4 and TSH should be monitored every 4 to 6 weeks after any change in L-thyroxine dosage (2, 69, 78). FT3 measurements are not useful in monitoring treatment because this test may be normal despite low FT4 and high TSH levels (55).

In the first months of life, it is essential to be aware of possible prolonged hyperthyroidism, which may be associated with premature craniosynostosis (69). It is also worth mentioning that patients who experience four or more episodes of insufficiently suppressed TSH (>5 U/mL) after 6 months of age have often shown poor academic performance later in life (105). Height and weight should be monitored regularly during check-ups. Periodic thyroid ultrasound is recommended in children with thyroid *in situ* (2). Delayed bone age usually becomes compatible with actual age by the third year of life (56).

Neurodevelopmental delay is rare in patients with CH treated from the first month of life (106). However, mild difficulties in motor skills and impairment of visuospatial processing and selective memory may be observed, presumed signs of poorly compensated fetal hypothyroidism (107–110). Furthermore, adequately treated children with CH show no differences in school performance compared to healthy children (111, 112). Despite early and adequate treatment, some children and adolescents with CH present mild and subclinical hearing deficits, mostly bilateral, of sensorineural type, involving high or very high frequencies (113, 114). Therefore, it is useful to periodically evaluate the psychomotor development and the school progression of children with CH, particularly in the areas of language, attention, memory, and behavior. In children with psychomotor delay, syndromic conditions and causes of intellectual disability must be excluded.

## Transient form of congenital hypothyroidism

Although hypothyroidism due to dysgenesis is usually permanent, approximately 35% of patients with a gland *in situ* have transient disease and will not require permanent treatment (115).

If permanent hypothyroidism has not been confirmed, it is advisable to discontinue therapy after 3 years. Treatment discontinuation guidelines include treatment suspension for a period of 4 weeks with a reassessment of thyroid function: if TSH is within reference limits with a normal FT4 level, transient CH is confirmed. Conversely, if TSH is marginally elevated but less than 10 mU/L, reassessment after 4 to 8 weeks is recommended (2, 78). If normal, transient hypothyroidism is assumed, and treatment can be permanently discontinued; conversely, if the TSH is elevated (>10 mU/L) and/or FT4 is low, treatment should be resumed (69, 116). However, treatment discontinuation should be limited to infants with a normally developed or enlarged thyroid showing normal TSH levels during replacement therapy (75).

Transient CH often has an environmental or iatrogenic origin (82). This type of CH may be related to iodine deficiency or excess, usually due to radiographic contrast agents or the application of iodinated disinfectants on the skin during hospitalization in the NICU (117). However, the widespread introduction of salt iodization programs has reduced the incidence of this type of transient CH. Other causes of transient CH are treatment with anti-thyroid drugs (methimazole, carbimazole, and propylthiouracil) and, rarely, the transplacental passage of antibodies that block the action of TSH (60). More than 50% of patients with *DUOX2* or *DUOX2A* variants present transient CH; heterozygous mutations in the *DUOX2* gene have recently been found in patients with transient CH (41, 118). Interestingly, only one case with a heterozygous missense variant in the *PAX8* gene presented with transient CH has been described (115). Finally, a transient form of CH is frequently found in premature infants (119). Preterm infants are at high risk of iodine deficiency due to the low iodine content in preterm infant formulas and parental nutrition (120).

It is not possible to distinguish the neonate with permanent CH from the neonate with transient CH at diagnosis. During treatment, however, some indicators such as low L-thyroxine requirement, low TSH level at diagnosis, and lack of increase in TSH during therapy may suggest a transient requirement (117). Currently, it is recommended to interrupt treatment if the daily dose of L-thyroxine is less than 25 µg (or <3 µg/kg/day) with stable or decreasing dosage requirements without an increase in TSH during therapy (2). Furthermore, a reassessment of thyroid function may be suggested as early as the 6^th^ month of life in children with glands *in situ* who require a dose of L-thyroxine <2-3 µg/kg/day (2, 78).

## Conclusions and future perspectives

Congenital hypothyroidism is one of the endocrine diseases that is changing the most compared to the past, thanks to both newborn screening and recent genetic advances. However, there are still open questions about the disease’s diagnosis, treatment, and management, which, 50 years later, require clear answers to improve the patients’ lives.

One of the unresolved issues concerns the extension of newborn screening to all countries globally, given that today, only 30% of countries are covered by this preventive practice. Therefore, it is imperative to find a way to extend newborn screening in areas not covered by this practice, especially in Asia and Africa, countries with a high birth rate, perhaps with pilot studies coordinated and managed by countries where screening is routinely performed.

Another open question concerns improvements in genetic analysis. Using NGS panels or whole exome sequencing will make identifying new genes and clinical phenotypes possible. Furthermore, the functional study of new genetic variants could broaden the classification of new pathogenic variants, describing new clinical phenotypes. Furthermore, epigenetics studies and environmental factors such as iodine could contribute to understanding the different penetrance and improve the genotype-phenotype correlation. Finally, improving treatment in daily clinical practice is necessary, identifying more appropriate dosages and more effective formulations, especially for paediatric patients, and, if possible, adapting therapeutic doses to the genotype. It is also necessary to re-discuss and conduct case-control studies to understand when to suspend treatment in patients with transient CH. Suspension should be based on chronological age or the dosage per kg of therapy, or both of these hypotheses should be evaluated based on genetic results. Therefore, implementing genetic diagnosis could have important clinical implications, allowing for individualized care in the future.
